# Potential Role of Drebrin A, an F-Actin Binding Protein, in Reactive Synaptic Plasticity after Pilocarpine-Induced Seizures: Functional Implications in Epilepsy

**DOI:** 10.1155/2012/474351

**Published:** 2012-02-14

**Authors:** Lotfi Ferhat

**Affiliations:** ^1^Aix-Marseille University, NICN, 13385 Marseille, France; ^2^CNRS, UMR 7259, 13385 Marseille, France

## Abstract

Several neurological disorders characterized by cognitive deficits, including Alzheimer's disease, down syndrome, and epilepsy exhibit abnormal spine density and/or morphology. Actin-based cytoskeleton network dynamics is critical for the regulation of spine morphology and synaptic function. In this paper, I consider the functions of drebrin A in cell shaping, spine plasticity, and synaptic function. Developmentally regulated brain protein (drebrin A) is one of the most abundant neuron-specific binding proteins of F-actin and its expression is increased in parallel with synapse formation. Drebrin A is particularly concentrated in dendritic spines receiving excitatory inputs. Our recent findings point to a critical role of DA in dendritic spine structural integrity and stabilization, likely via regulation of actin cytoskeleton dynamics, and glutamatergic synaptic function that underlies the development of spontaneous recurrent seizures in pilocarpine-treated animals. Further research into this area may provide useful insights into the pathology of status epilepticus and epileptogenic mechanisms and ultimately may provide the basis for future treatment options.

## 1. Introduction

The human brain is composed by hundred billion neurons interconnected in order to form functional neuronal networks that control higher brain functions, such as learning, thoughts, emotions, and memory throughout life. The communication between neurons within neuronal networks is mediated via synapses. Tight control mechanisms of the formation, growth, and connectivity of synapses are crucial for accurate neural network activity and normal brain function. For example, the development, remodeling, and elimination of excitatory synapses on dendritic spines represent ways of refining the microcircuitry in the brain. Thus, when processes involved in structural synapses and/or synaptic function go awry, either during normal aging or in disease, dysfunction of the organism occurs. 

## 2. Dendritic Spines and Functions

Dendritic spines are tiny protrusions from the dendritic tree that serve as the postsynaptic component for the vast majority of excitatory synapses in the central nervous system [1–4]. These protrusions are found on most excitatory and some inhibitory neurons [2, 3, 5, 6]. The dendritic spine consists of a bulbous head connected to the dendritic shaft by a narrow neck [1, 7]. The narrow neck of the spine forms a spatially isolated compartment where molecular signals can rise and drop without diffusing to neighboring spines along the parent dendrite, thus allowing the isolation and/or amplification of received signals. Such limitation of molecular signals to one spine may participate to the “axonal inputs specificity,” permitting a given set of axon terminals to induce alterations only within synapses that are specific to their postsynaptic contacts and not at other synapses on the same neuron formed by different axon terminals [3, 8]. Thus, it is widely accepted that dendritic spine constitutes a postsynaptic biochemical compartment that separates the synaptic space from the dendritic shaft and allows each spine to function as a partially independent unit [2, 9]. In addition to constitute sites for the development of glutamatergic neuronal networks, these dendritic protrusions might be cellular substrates for synaptic transmission and plasticity [3, 10].

Numerous studies have shown that spines are highly motile structures, and their shape, size, and density change during development and adulthood. During development, dendritic protrusions start out as filopodia, which evolve directly into dendritic spines or lead to the formation of shaft synapses from which spines rise at later stages of synaptogenesis [11–13]. In adults, these changes are influenced by several factors, including synaptic activity and plasticity [14–16], and are also associated with learning [17], aging [18], as well as diseases. Indeed, abnormal changes in spine density and morphology are observed in many neurological disorders characterized by cognitive deficits, such as Alzheimer's disease (AD), down syndrome, fragile X syndrome, and epilepsy [2, 3, 19]. Because spine morphology is closely associated with synaptic function, altered spines in disease conditions are likely to have diverse functional effects leading to the neurological symptoms of such disorders. The molecular mechanisms by which physiological and pathological stimuli modulate dendritic spine structure and function are not fully understood, but may involve regulation of the actin cytoskeleton [3, 4, 20].

## 3. Dendritic Spines and Actin Cytoskeleton

The actin filament (F-actin) is one of the most abundant cytoskeleton elements found in dendritic spines [21–24]. These actin filaments are thought to be the most convincing key site for the molecular mechanisms regulating spine plasticity [4, 25–28]. In addition, time-lapse studies showed that actin-based plasticity in dendritic spines is activity-dependent [27]. Consistent with this observation, it has been shown that long-term potentiation (LTP), a well-described form of experimental synaptic plasticity, is associated with enhanced F-actin content in dendritic spines *in vivo* [29] and *in vitro* [30, 31]. Therefore, the identification of the molecular basis underlying the spine plasticity and function are fundamental to understand the mechanisms of synaptic plasticity in physiological conditions as well as in some neurological disorders.

## 4. Drebrin A in Dendritic Spine Plasticity and Synaptic Function

**Figure 1:**
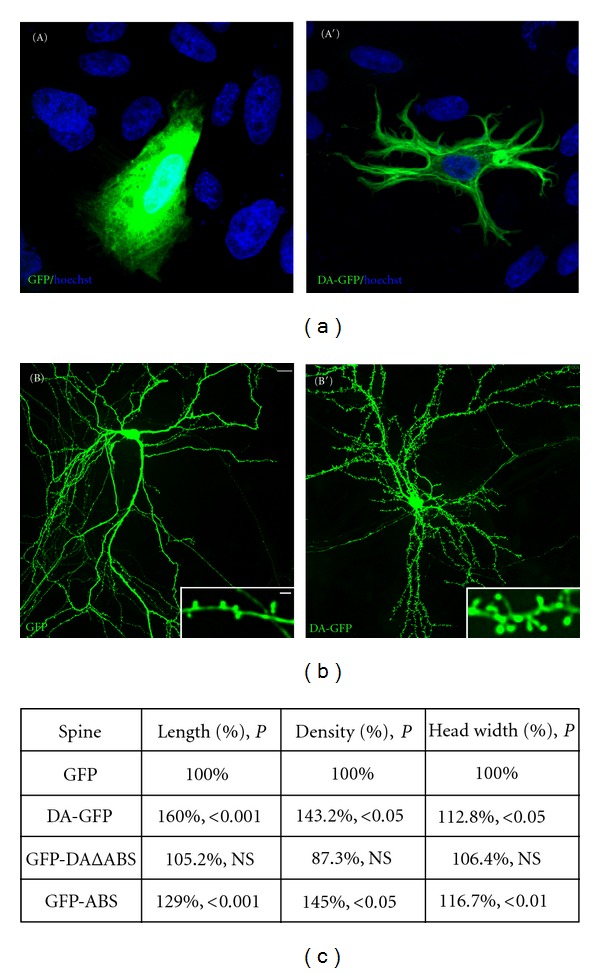
Drebrin A overexpression affects the morphology of cultured CHO-K1 cells and dendritic spines plasticity of cultured mature hippocampal neurons. In contrast to GFP (A), CHO-K1 cells transfected with DA-GFP (A′) display striking morphological changes characterized by the formation of several processes frequently branched. Blue color reveals nuclear staining by Hoechst 33258. Mature hippocampal neurons were transfected at 21 days *in vitro* with GFP (B) and DA-GFP (B′). After 2 days of transfection (23 days *in vitro*), neurons were fixed and then examined by a confocal microscope. Striking morphological changes are observed between dendritic spines of GFP- and those of DA-GFP neurons. Indeed, the dendrites of DA-GFP neurons display longer spines (inset in B′) compared with those found in GFP neurons (inset in (B)). Some spines labeled with DA-GFP can reach over 5 *μ*m (inset in (B′), see asterisk). Scale bars: 5 *μ*m in (A), (A′), (B), and (B′) and 2 *μ*m in insets. (c) Table showing the spine length, density, and head width of GFP, DA-GFP, GFP-DAΔABS, and GFP-ABS neurons. GFP: green fluorescent protein; DA-GFP: drebrin A fused to GFP; GFP-DAΔABS: drebrin A without its actin binding site (ABS) fused to GFP; GFP-ABS: actin binding site of drebrin A fused to GFP; *P*: probability; NS: not significant.

Several proteins that bind to actin filaments govern the actin cytoskeleton properties. The adult isoform of drebrin, drebrin A (DA), a major neuron-specific binding protein of F-actin, emerges as a convincing candidate protein for providing particular characteristics to the actin cytoskeleton of dendritic spines [32–35]. DA is specifically and highly enriched in dendritic spines of mature neurons [36–39] and is shown to inhibit the actin-binding activity of tropomyosin, fascin and *α*-actinin [40, 41]. *In vitro*, DA also blocks the interaction between actin and myosin [36, 42], indicating that it modulates actin filament contractility. In fibroblasts, the overexpression of DA causes reorganization and stabilization of actin filaments leading to the alteration in their cell morphology [35, 38, 43] (see Figure 1(a)), and that these effects are mediated by its actin-binding domain [35, 38]. Such transfections in mature hippocampal neurons revealed that DA increases dendritic spine length, size, and density [35, 38] (see Figures 1(b) and 1(c)), and again these effects require the actin-binding domain of DA [35, 38]. Conversely, the reduction of DA expression by antisense oligonucleotide treatment in developing hippocampal neurons significantly decreases the width and density of filopodia spines [44, 45]. Overall, these observations strongly suggest that DA regulates the physiological dendritic spine plasticity via regulation of actin cytoskeleton reorganization and dynamics. In addition to its role in cell shape and dendritic spine plasticity, DA might play a role in regulating synaptic function. Indeed, our electrophysiological data revealed that overexpression of DA in cultured mature hippocampal neurons increases excitatory and inhibitory synaptic transmission leading to the alteration of the normal excitatory-inhibitory (E/I) balance in favor of excitation [35, 38]. 

## 5. Drebrin A in Reactive Synaptic Plasticity

**Figure 2:**
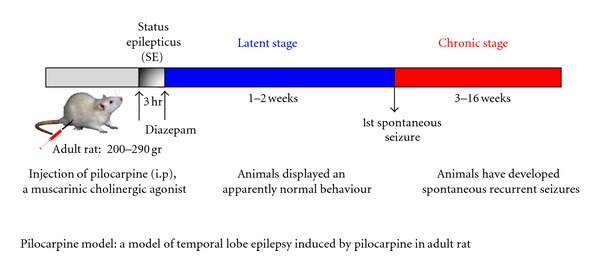
Scheme illustrating a model of temporal lobe epilepsy induced by pilocarpine in adult rat. Adult rats were injected intraperitoneally (i.p.) with pilocarpine hydrochloride, a muscarinic cholinergic agonist. The injection produces a status epilepticus (SE) that is stopped after 3 hr by a single injection of diazepam to reduce mortality of the animals. The rats were then observed periodically in the vivarium for general behavior and occurrence of spontaneous seizures for a period of 16 weeks. Pilocarpine-treated animals were analyzed at several postinjection intervals: during the latent period, when animals displayed an apparently normal behavior (1 and 2 weeks), and during the chronic stage, when the animals have developed spontaneous recurrent limbic seizures (8–16 weeks). It has been previously demonstrated in this model of pilocarpine-treated rats, by using *in vivo* electroencephalographic recordings that the first spontaneous seizures occur during the third week after status epilepticus [46]. In this model, a dynamic reorganization of the glutamatergic network, including neurodegeneration, neurogenesis, neo-spinogenesis, spine morphogenesis, and neo-synaptogenesis associated with an aberrant sprouting of granule cell axons, is well established in the dentate gyrus.

Some of the molecular mechanisms that are involved in spine plasticity under physiological conditions could also be reused in the disease states, but may be activated in an extreme or inappropriate manner leading to the pathological changes in dendritic structure and dynamics. Thus, we hypothesized that DA is one of the regulators of actin filaments during the physiopathological conditions such as reactive dendritic spine plasticity. Since a thorough analysis of the molecular mechanisms underlying synaptic dysfunction in various neurological disorders is difficult to perform using *postmortem* human tissue, several laboratories have produced animal models that mimic some symptoms of a particular neurological disorder. For this purpose, we tested our hypothesis, relying on a well-characterized experimental model of temporal lobe epilepsy (TLE) induced by pilocarpine in adult rats (see Figure 2). This model was selected because a dynamic reorganization of the glutamatergic network, including neurodegeneration [47–50], neurogenesis [51–53], neo-spinogenesis, morphogenesis [54, 55], and neo-synaptogenesis associated with an aberrant sprouting of granule cell axons [47, 56, 57], is well established in the dentate gyrus (DG). This reactive plasticity contributes to the dentate granule-cell hyperexcitability that could lead to the emergence of recurrent spontaneous seizures. The dynamic reorganization begins after the initial period of status epilepticus following pilocarpine injection, continues during the silent period when animals display a normal behavior, and reaches a plateau at the chronic stage when the animals have developed spontaneous recurrent seizures (see Figure 2). Altogether, these data indicate that in pilocarpine-induced seizures, DA is not critical for spinogenesis and morphogenesis but is rather involved in the structural integrity and stabilization of dendritic spines of hippocampal granule cells. This likely occurs via regulation of the actin cytoskeleton dynamics, and glutamatergic synaptic function that underlies the development of recurrent spontaneous seizures described in the pilocarpine model [39]. 

### 5.1. Drebrin A May Be Involved in the Structural Integrity and Stabilization of Dendritic Spines at a Chronic Stage of Epilepsy

Under physiological conditions, mossy cells, the major type of neurons within the hilar region of the hippocampal DG, receive excitatory inputs on their characteristic “thorny excrescences” from mossy fiber axons of the dentate granule cells [59–61]. Thorny excrescences are predominantly present on the proximal dendrites of cells displaying a mossy appearance, hence their name [62]. Mossy cells in turn send their axonal projections to the ipsi- and contra-lateral inner molecular layer (IML) and form excitatory synapses mainly onto proximal dendritic spines of granule cells [61, 63–67]. It has been reported that spiny hilar mossy cells and their axon terminals degenerate in the human TLE and in the pilocarpine model [48, 57]. The degeneration of mossy cells and their axon terminals after pilocarpine-induced seizures results in a deinnervation of their postsynaptic targets, granule cell dendrites within the IML. Several other experimental paradigms such as entorhinal cortex lesion or hippocampal deafferentation with severe loss of presynaptic input cause alterations of postsynaptic target structures, including a loss of dendritic spines [68–71]. According to our results, one of the effects of this deinnervation is a significant decrease in the immunolabeling of Bassoon, a specific marker of presynaptic active zones, in the IML 1-2 weeks after pilocarpine treatment [39]. The subsequent recovery of Bassoon immunolabeling in the IML 12 weeks after pilocarpine treatment is likely due to the formation of aberrant sprouting of mossy fibers after status epilepticus [47, 56, 57], which has been shown to be involved in the establishment of functional excitatory synaptic boutons on granule cell dendrites [57, 72, 73]. If the recovery in Bassoon immunolabeling at chronic stage reflects an increased number of synaptic terminals due to mossy fiber sprouting, then we would expect a significant reexpression of DA in granule cell dendritic spines associated with newly formed synapses. Indeed, our data clearly show an increase in the expression of DA protein which coincides with an increase in Bassoon-containing terminals at 12 weeks after pilocarpine injection, further suggesting that the increase in DA protein levels occurs in dendritic spines that are associated with newly formed synapses.

**Figure 3:**
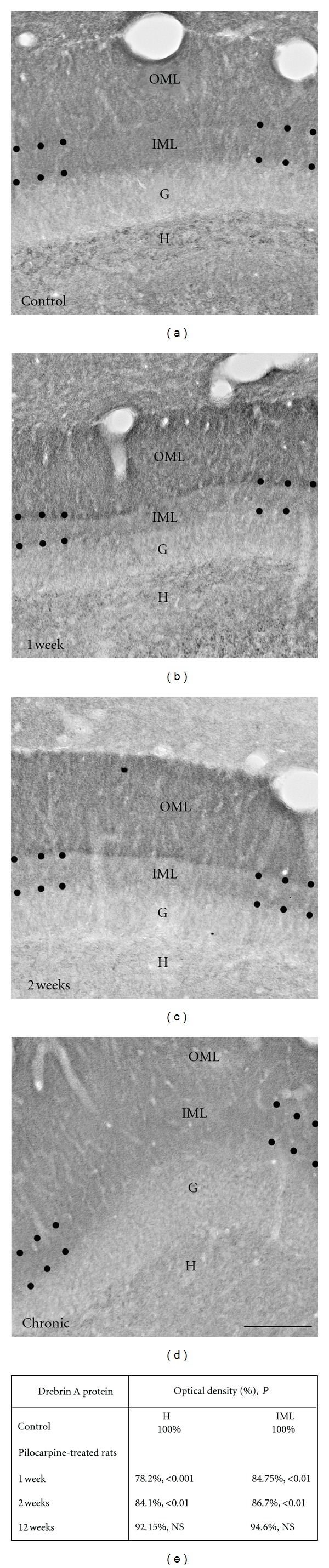
Comparison of immunohistochemical labeling for drebrin A in hippocampal formation from control (Ctl; (a)) and pilocarpine-treated animals at 1 week (b), 2 weeks (c), and 12 weeks (d). The major difference compared to Ctl animals (a) is observed in the molecular layer (M) and in the hilus (H). In the pilocarpine-treated rats at 1 week (b) and 2 weeks (c), DA immunolabeling is strongly decreased in the inner molecular layer (IML) and in the H when compared with Ctl rats (a), whereas no difference is observed in pilocarpine-treated animals at 12 weeks (Chronic; (d)). The loss of labeling in these regions contrasted with the preservation of the levels of immunolabeling in the outer molecular layer (OML) and granule cell layer (G). Scale bars = 50 *μ*m in ((a), (b), (c), and (d)). (e) Table showing the comparison of % optical density for drebrin A protein in H and IML between control and pilocarpine-treated animals at 1, 2, and 12 weeks. H: hilus; IML: inner molecular layer; *P*: probability, NS: not significant. Modified with permission from [39].

**Figure 4:**
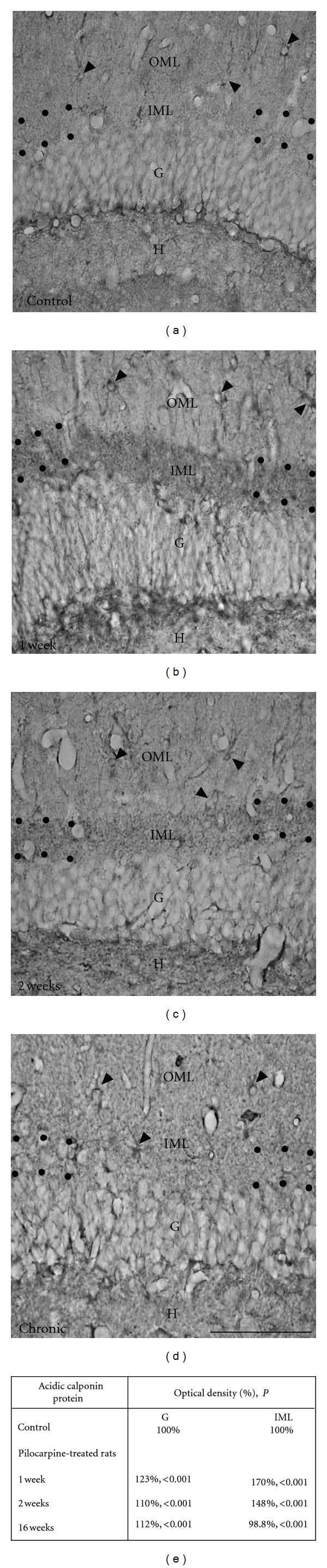
Comparison of immunohistochemical labeling for acidic calponin in hippocampal formation from control (Ctl; a) and pilocarpine-treated animals at 1 week (b), 2 weeks (c), and 16 weeks (d). In a Ctl rat (a), immunolabeling for acidic calponin in the dentate gyrus (DG) is mainly found in astrocytes (arrows) and cells located along the infragranular region of the granule cell layer (G). A diffuse staining is evenly observed in all parts of the molecular layer (M) including the inner molecular layer (IML). In pilocarpine-treated animals at 1 and 2 weeks, immunolabeling for acidic calponin is substantially increased in IML of the DG compared with Ctl (a) and to pilocarpine-treated rats at 16 weeks (d). In all pilocarpine-treated animals, immunolabeling for acidic calponin is also found in astrocytes (see arrows) located in the M. Scale bars = 50 *μ*m in (a), (b), (c), and (d). (e) Table showing the comparison of % optical density for acidic calponin protein in G and IML between Ctl and pilocarpine-treated animals at 1, 2, and 16 weeks. G: granule cell layer; IML: inner molecular layer; *P*: probability; NS: not significant. Modified with permission from [58].

In addition to the degeneration of mossy cells and their synaptic inputs in the IML, dendrites of dentate granule cells display a global spine loss immediately after the status epilepticus induced by injection of pilocarpine. This spine loss is transitory and is followed by a recovery in spine density that begins 3 days after status epilepticus and reaches a plateau level 15–35 days later. Conversely, these spine densities are still low in comparison with control values [54, 55, 74]. Interestingly, the recovery proportion depends on spine morphology. Indeed, mushroom-shaped spines recover slower, and partially, than thin spines bearing a clear neck [54, 55]. Based on our results, one of the effects of this spine loss consists in a significant reduction of DA immunolabeling observed in the IML 1 and 2 weeks after pilocarpine treatment, when spinogenesis and spine morphogenesis occur (see Figure 3). This finding is consistent with the work of Takahashi and colleagues [44, 45], showing that downregulation of DA by antisense oligonucleotide treatment significantly decreases the density of filopodia spines. Therefore, our data indicate that DA is not crucial in the recovery of these plastic changes in spine shape and density after the initial acute seizures-induced by pilocarpine, in contrast to other actin-binding proteins such as acidic calponin [58]. Indeed, several lines of evidence reinforce this idea based on the following observations: (1) in mature neurons, acidic calponin is localized mostly in dendritic spines [75]; (2) overexpression of acidic calponin in cultured HEK 293 cells induces major morphological changes through a reorganization of actin filaments [76]; (3) such transfections in primary cultures of rat hippocampal neurons causes an elongation of spines and an increase of their density [77]; (4) the increase in the immunolabeling for acidic calponin is observed at the latent period (1-2 weeks after pilocarpine injection) [58] (see Figure 4), a period of important remodeling of dendritic spine shape and density in dentate granule cells [54, 55]; finally, the main *in vitro* effect of the calponin family is to inhibit actomyosin activity [78, 79]. Altogether, these observations indicate that acidic calponin may affect the organization and the dynamics of actin filaments, leading to the plasticity in the shape and density of dendritic spines after status epilepticus.

The fact that the recovery of DA protein expression is observed at the chronic stage (see Figure 3), when functional glutamatergic synapses are being established may indicate a critical role of DA in the structural integrity and stabilization of dendritic spines and synaptic function at this period. In addition to DA, other actin-binding proteins such as synaptopodin may contribute to these functions. In favor of this idea, the work of Roth et al. [80] showed that the expression of synaptopodin protein, also enriched in dendritic spines, is induced at the chronic stage and is associated with synaptic remodeling processes following kainate-induced epilepsy in rat. Nevertheless, we cannot completely exclude the possibility that the subsequent recovery of DA protein expression at the chronic stage after transient reduction reflects in part the plastic changes in spine shape and density induced by DA on outgrowing dendrites of newly formed granule cells subsequent to pilocarpine-induced neurogenesis [51–53]. This hypothesis is supported by recent data [35, 38], showing that overexpression of DA in mature hippocampal neurons induces a significant increase in spine length, size, and density. These spine plastic changes might be related to the properties of the drebrin family, which is shown to stimulate polymerization via profilin [81–83], bundling, and stabilization of actin filaments [35, 38, 84, 85]. Therefore, it seems that the period of spinogenesis on dendrites of newly formed granule cells coincides with the period when the mossy fiber sprouting reaches a plateau level [47, 56, 57], suggesting that these new spines might be involved in the formation of aberrant functional synapses with newly formed mossy fiber terminals. Indeed, our data showed that the increase in DA protein levels in the IML at chronic stage occurs within dendritic spines that are adjacent to terminals labeled for vGlut1, a glutamatergic presynaptic marker, and Bassoon. Altogether, our observations suggest that the main part of DA recovery in the IML at chronic stage occurs in new spines located on preexisting granule cell dendrites.

### 5.2. Molecular Mechanisms Mediating Drebrin A Loss at a Latent Stage of Epilepsy

**Figure 5:**
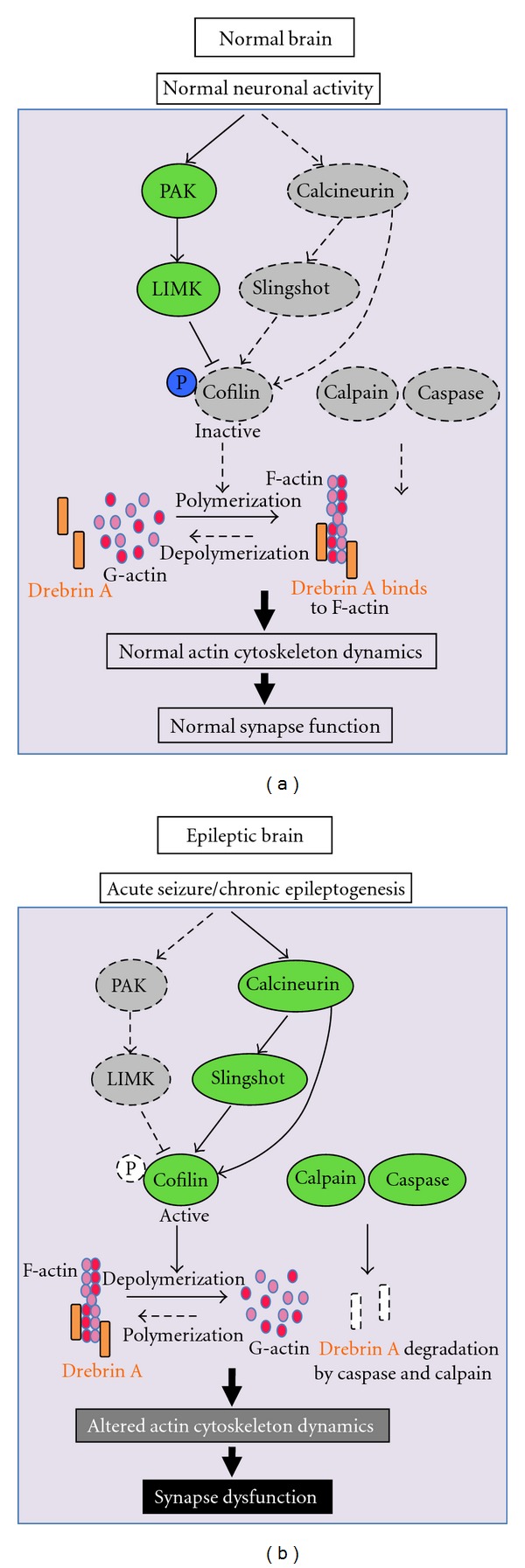
Potential signaling pathways and molecular mechanisms mediating dendritic spine injury and drebrin A loss in epilepsy. Acute seizure or chronic epileptogenesis may lead to activation of calcium-dependent phosphatase, calcineurin, which in turn causes cofilin dephosphorylation either directly or indirectly via an intermediary phosphatase, known as slingshot. Cofilin activity can also be regulated by phosphorylation via the PAK-LIM-kinase pathway. Thus, higher slingshot and/or lower PAK/LIM kinase activities in the hippocampi with epilepsy, leading to less phosphorylation of cofilin, would potentially increase cofilin binding to F-actin, and this could cause depolymerization of F-actin, leading to the prevention and/or dissociation of drebrin from its actin-binding site. This would result in drebrin A translocation to the cytosol leading to its degradation by active caspase. Ultimately, the breakdown of the actin cytoskeleton in dendrites can lead to synaptic dysfunction in epilepsy.

At least three molecular mechanisms have been considered to explain the loss of DA in pilocarpine-treated animals. First, drebrin has been characterized as a substrate of caspase-6 [86], which is activated in epilepsy [87–90]. Thus, direct breakdown by caspases is a possible reason for the failure to detect drebrin immunoreactivity in the IML. Second, the cleavage of cytoskeletal proteins such as fodrin [91], which is observed in epilepsy, could lead to the release of drebrin from the membrane compartment into the cytosol. This view is supported by a protease such as calpain, which might well be involved [91]. A third potential mechanism involves the activation of a calcium-dependent phosphatase, calcineurin (CaN), via its regulation of the actin-depolymerizing factor, cofilin [92, 93]. Cofilin is an actin-binding protein, which when dephosphorylated binds to F-actin and causes its depolymerization [94, 95]. Recent studies indicated that CaN induces cofilin dephosphorylation either directly [96] or indirectly via the slingshot phosphatase [93, 97–99]. Cofilin activity can also be regulated by phosphorylation via the PAK-LIM-kinase pathway [93, 100, 101]. In all cases, higher slingshot and/or lower PAK/LIM kinase activities in the hippocampi with epilepsy leading to a decrease in cofilin phosphorylation, would potentially increase cofilin binding to F-actin, and this could prevent and/or dissociate drebrin from its actin-binding site. As a result, drebrin would be translocated to the cytosol and degraded by caspase [86–90, 102] and/or by calpain, because of its several calpain cleavage sites enriched in proline, glutamate, serine, and threonine (PEST sites) [36, 86, 103]. This idea is supported by *in vitro* data obtained from cultured hippocampal neurons treated with soluble A*β*1-42 oligomer as well as *in vivo* experiments in which intracerebral injections of PAK inhibitors in rodents induce translocation of drebrin from the membrane to the cytosol [104]. Ultimately, the alteration of the actin cytoskeleton dynamics in dendrites can lead to synaptic dysfunction in epilepsy [92, 93, 105] (see Figure 5). Obviously, all these molecular mechanisms can take place simultaneously to participate in drebrin loss and dendritic injury in the IML of pilocarpine-treated animals.

### 5.3. Drebrin A May Be Involved in Synaptic Function at a Chronic Stage of Epilepsy: Functional Implications in Epilepsy

Besides its role in cell shape and dendritic spine plasticity, DA may play a role in synaptic function. Indeed, it has been shown that DA induces spinous clustering of the post-synaptic density (PSD) scaffold protein, PSD-95 [44] as well as activity-dependent synaptic targeting of NMDA receptors [45]. Upon induction of LTP in the hippocampus, drebrin expression is enhanced within dendritic spines [29]. Consistent with this observation, we showed that the recovery in DA protein levels in the IML occurs within dendritic spines that are involved in the formation of aberrant functional glutamatergic synapses with the newly formed mossy fiber terminals of presumed granule cells. In addition, the reduction of DA mediated by antisense oligonucleotides causes cognitive deficits [106]. Recently, we reported the functional role of DA in regulating synaptic transmission. Indeed, the overexpression of DA induces an increase in glutamatergic but not GABAergic synapses and results in the alteration of the normal excitatory-inhibitory (E/I) ratio in favor of excitation in mature hippocampal neurons [35, 38]. As epilepsy involves hyperexcitable neurons, a basic assumption links the pathogenesis of epilepsy and the generation of synchronized neuronal activity with an imbalance between inhibitory and excitatory neurotransmission in favor of the latter [107, 108]. Thus, we propose that DA may serve as one of the molecular factors leading to the alteration of the normal excitatory-inhibitory balance in favor of excitation observed in the DG at the chronic stage of epilepsy [109]. In this context, our studies identified DA as a potential target in order to modulate hyperexcitability in epilepsy.

## 6. Conclusions

**Figure 6:**
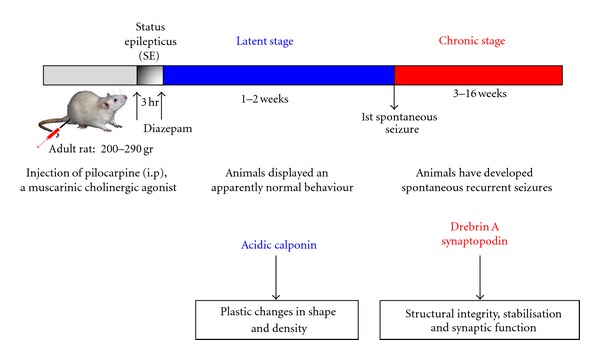
Scheme illustrating the potential role of drebrin A in reactive synaptic plasticity after pilocarpine-induced seizures. Drebrin A together with other proteins such as synaptopodin might be more involved in the structural integrity and stabilization of dendritic spines of hippocampal granule cells, and glutamatergic synaptic function that underlies the development of spontaneous recurrent seizures in pilocarpine animals, at chronic stage, whereas acidic calponin could contribute to the plastic changes in shape and density occurring after status epilepticus.

Based on all these findings, we conclude that DA together with other proteins such as synaptopodin might be more involved in the structural integrity and stabilization of dendritic spines. These effects are probably mediated via regulation of actin cytoskeleton dynamics, and glutamatergic synaptic function that underlies the development of spontaneous seizures in pilocarpine animals at chronic stage. In contrast, acidic calponin could contribute to the plastic changes in shape and density occurring after a status epilepticus described in pilocarpine model [54, 55, 92] (see Figure 6). Further insights into the mechanisms how actin-based spine plasticity is induced by seizures could have a major impact in preventing the long-term negative consequences of epilepsy and ultimately may provide the basis for future treatment options.
